# Finnish Diabetes Risk Score Is Associated with Impaired Insulin Secretion and Insulin Sensitivity, Drug-Treated Hypertension and Cardiovascular Disease: A Follow-Up Study of the METSIM Cohort

**DOI:** 10.1371/journal.pone.0166584

**Published:** 2016-11-16

**Authors:** Maria Fizelova, Raimo Jauhiainen, Alena Stančáková, Johanna Kuusisto, Markku Laakso

**Affiliations:** 1 Institute of Clinical Medicine, Internal Medicine, University of Eastern Finland, Kuopio, Finland; 2 Institute of Clinical Medicine, Internal Medicine, University of Eastern Finland and Kuopio University Hospital, Kuopio, Finland; Virgen Macarena University Hospital, School of Medicine, University of Seville, SPAIN

## Abstract

We investigated the association of the Finnish Diabetes Risk Score (FINDRISC) with insulin secretion, insulin sensitivity, and risk of type 2 diabetes, drug-treated hypertension, cardiovascular (CVD) events and total mortality in a follow-up study of the Metabolic Syndrome in Men (METSIM) cohort. The METSIM study includes 10,197 Finnish men, aged 45–73 years, and examined in 2005–2010. Of 8,749 non-diabetic participants of the METSIM study 693 developed incident type 2 diabetes, 225 started antihypertensive medication, 351 had a CVD event, and 392 died during a 8.2-year follow-up. The FINDRISC was significantly associated with decreases in insulin secretion and insulin sensitivity (*P*<0.0001), and with a 4.14-fold increased risk of incident type 2 diabetes, 2.43-fold increased risk of drug-treated hypertension, 1.61-fold increased risk of CVD, and 1.55-increased risk of total mortality (the FINDRISC ≥12 vs. < 12 points). In conclusion, the FINDRISC predicts impairment in insulin secretion and insulin sensitivity, the conversion to type 2 diabetes, drug-treated hypertension, CVD events and total mortality.

## Introduction

Early detection of individuals at high risk of the development of type 2 diabetes is of great importance [1–3]. Therefore, a variety of risk scores has been developed and applied either in the cross-sectional [4] or prospective setting to evaluate the risk of diabetes [5,6]. These risk scores include conventional risk factors for diabetes, including age, body mass index (BMI), information on diet and family history [6,7], laboratory measurements [8,9] and/or genetic information [10,11].

The Finnish Diabetes Risk Score (FINDRISC) is a simple and practical tool to identify people at high risk for type 2 diabetes [12]. The FINDRISC is composed of eight easily available parameters (age, BMI, waist circumference, hypertension, physical activity, diet, history of hyperglycemia, and family history of diabetes). The FINDRISC has been shown to predict not only type 2 diabetes [13], but also coronary heart disease, stroke and total mortality [13].

Insulin resistance and impaired insulin secretion are the two main pathophysiological mechanisms leading to type 2 diabetes [14]. Insulin resistance precedes and predicts diabetes [15], and clusters with several cardiovascular risk factors such as obesity, hypertension, and dyslipidemia [16]. Additionally, insulin resistance is an important risk factor for CVD [17]. A simplified version of the FINDRISC has been linked with insulin resistance in two small cross-sectional studies [18,19] but no large population-based prospective studies are available where the FINDRISC had been evaluated as a predictor for changes in insulin secretion, insulin resistance or insulin resistance-related traits.

Given the fact that impaired insulin secretion is needed for the conversion to diabetes [20] we hypothesized that the FINDRISC is also a marker of impaired insulin secretion and insulin resistance related traits. To this aim we investigated the association of the FINDRISC with insulin secretion, insulin sensitivity, insulin resistance related traits, incident type 2 diabetes, drug-treated hypertension, CVD events and total mortality in a 6-year follow-up of a large Finnish population-based study.

## Materials and Methods

### Subjects and clinical measurements

The Metabolic Syndrome in Men (METSIM) includes 10,197 Finnish men, aged from 45 to 74 years (mean±SD 58±7 years; mean body mass index (BMI) 27±4 kg/m^2^), and randomly selected from the population register of Kuopio, Eastern Finland (population 105,000). The cross-sectional study was performed in 2005–2010. Each participant had one-day outpatient visit to the Clinical Research Unit, University of Eastern Finland, Kuopio, Finland. The study has been described in detail elsewhere [21]. The present report includes 8,749 non-diabetic participants of the METSIM baseline study. Glucose tolerance was evaluated with a 2-hour oral glucose tolerance test (OGTT; 75g of glucose) after 12h overnight fast. Glucose tolerance status was classified according to the American Diabetes Association (ADA) criteria [22]. Among the participants 3,034 (34.7%) had normal glucose tolerance, 4,344 (49.7%) isolated impaired fasting glucose (IIFG), 312 (3.6%) isolated impaired glucose tolerance (IIGT), and 1,059 (12.1%) both IFG and IGT. Participants with type 1 diabetes (N = 25), previously diagnosed type 2 diabetes (N = 763), type 2 diabetes diagnosed at baseline (N = 649), or with missing OGTT data (N = 11) were excluded from the analyses.

The prospective ongoing follow-up study started in 2010 and so far 5,552 non-diabetic individuals have been re-examined (mean follow-up time of 4.6-years; range of follow-up time 0.83–9.4 years). A total of 5,401 participants were included in current statistical analyses (17 participants had missing OGTT data, and 134 participants developed diabetes between the baseline and follow-up visits).

#### Ethics Statement

The study was approved by the Ethics Committee of the University of Eastern Finland and Kuopio University Hospital and was conducted in accordance with the Helsinki Declaration. All study participants provided written informed consent.

### The Finnish Diabetes Risk Score (FINDRISC)

The FINDRISC data were available for 8,745 non-diabetic participants at baseline. A total of 8 variables are included in the FINDRISC (age, BMI, waist circumference, physical activity (at least 30 min of physical activity daily at work and/or during leisure time), diet (daily consumption of vegetables, fruits or berries), antihypertensive drug treatment, history of hyperglycemia, and family history of diabetes. The maximal score observed was 26 points (mean score 10.4±4.9). The participants were divided into the four categories based on the FINDRISC scale (category 1, low-risk, FINDRISC <7, N = 2,011; category 2, slightly elevated risk, FINDRISC 7–11, N = 3,302; category 3, moderate risk, FINDRISC 12–14, N = 1,615; category 4, high and very high risk, FINDRISC >15, N = 1,817) proposed by Dr. Jaakko Tuomilehto *et al*. (http://www.diabetes.fi/files/502/eRiskitestilomake.pdf).

### Definitions of incident type 2 diabetes, drug reimbursement for hypertension, cardiovascular (CVD) events and total mortality

Among the 8,749 non-diabetic men at baseline, 693 developed incident type 2 diabetes between the baseline study and 30^th^ of June 2016. Diagnosis of new-onset type 2 diabetes was based on: 1) fasting plasma glucose (FPG) ≥7.0 mmol/L, 2-hour plasma glucose (2hPG) in an OGTT ≥11.1 mmol/L or glycated hemoglobin (HbA1c) ≥6.5% (395 new cases of diabetes) among nondiabetic individuals who participated in the ongoing follow-up study, or 2) antidiabetic medication started between the baseline study and follow-up study (261 new cases of diabetes; information obtained from the National Drug Reimbursement registry for all 8,749 nondiabetic participants), or 3) type 2 diabetes diagnosed by a physician based on medical records and/or FPG ≥7.0 mmol/L, 2hPG≥11.1 mmol/L or HbA1c ≥6.5% in outpatient/primary care laboratory measurements (N = 37 new cases of type 2 diabetes).

A total of 225 non-diabetic participants started a new antihypertensive treatment between the baseline and follow-up studies on the basis of the National Drug Reimbursement registry (data available until December 31, 2013). Non-diabetic participants receiving antihypertensive treatment (N = 1,537) started prior to the baseline study were excluded from statistical analyses when analyzing the associations of baseline variables with the development of drug-treated hypertension.

An incident CVD event was defined as non-fatal myocardial infarction, coronary heart disease death, or fatal and nonfatal cerebral infarction which occurred between the baseline and follow-up studies (data available until June 30, 2015). CVD events were defined according to the internationally accepted criteria [23,24] and verified from the hospital records. Non-diabetic individuals with non-fatal myocardial infarction (N = 344) and stroke (N = 194) before the baseline were excluded from statistical analyses.

Out of 8,749 non-diabetic participants a total of 392 died during the follow-up. The information on mortality was obtained from the Finnish Mortality registry.

### Clinical and laboratory measurements

Height was measured without shoes to the nearest 0.5 cm. Weight was measured in light clothing with a calibrated digital scale (Seca 877, Hamburg, Germany), and rounded up to the nearest 0.1 kg. BMI was calculated as weight (kg) divided by height squared. Waist was measured to the nearest 0.5 cm as the average of two measurements taken after inspiration and expiration at the midpoint between the lowest rib and iliac crest. Hip circumference (at the level of the trochanter major) was measured to the nearest 0.5 cm. Body composition was determined by bioelectrical impedance (Bioimpedance Analyzer Model BIA 101, Akern SrL, Florence, Italy) in subjects in the supine position after a 12-h overnight fast. Three measurements of blood pressure (interval 1.5 min) were performed in the sitting position after a 10-min rest with mercury sphygmomanometer. The average of three measurements was used to calculate systolic and diastolic blood pressure. Physical activity refers to leisure time physical activity (physically active, regular exercise at least 30 min ≥1 times per week vs. physically inactive, occasional exercise or no exercise). Smoking status was defined as current smoking (yes vs. no). Plasma glucose was measured by an enzymatic hexokinase photometric assay (Konelab Systems reagents, Thermo Fisher Scientific; Vantaa, Finland). Insulin was determined by immunoassay (ADVIA Centaur Insulin IRI no. 02230141; Siemens Medical Solutions Diagnostics, Tarrytown, NY). HbA_1c_ was analyzed by high-performance liquid chromatography (HPLC) using Tosoh G7 glycohemoglobin analyzer (Tosoh Bioscience, Inc. San Francisco, CA, USA) and calibrated according to the DCCT Standard. Total triglycerides (TGs), plasma free fatty acids (FFAs), high-density lipoprotein (HDL) cholesterol, and low-density lipoprotein (LDL) cholesterol were measured by enzymatic colorimetric tests (Konelab Systems Reagents, Thermo Fisher Scientific; Vantaa, Finland). Plasma adiponectin was measured by an ELISA (human adiponectin ELISA kit; Linco Research), and alanine aminotransferase by enzymatic photometric test (Konelab Systems reagent). Apolipoproteins A1 and B (ApoA1 and ApoB) were quantified by immunoturbidimetry (Konelab Systems Reagents).

### Calculations

The trapezoidal method was used to calculate the glucose area under the curve (Glucose AUC) based on the OGTT samples collected at 0, 30, and 120 min. Matsuda insulin sensitivity index [25], and early-phase insulin secretion (InsulinAUC_0-30_/GlucoseAUC_0-30_) [21] were calculated as previously described. Disposition index (a marker of insulin secretion) was calculated as Matsuda ISIxInsulinAUC_0-30_/GlucoseAUC_0-30_. The Cockcroft-Gault equation (140—age) x weight x 1.2278/serum creatinine concentration) was used to estimate glomerular filtration rate (eGFR).

### Statistical analysis

Statistical analyses were conducted using IBM SPSS version 19 (SPSS, Chicago, IL). All variables (except for age) were logarithmically transformed for statistical analyses due to their skewed distributions. One-way ANOVA was used to evaluate the differences in metabolic and clinical characteristics across the categories of the FINDRISC. ANCOVA was used to adjust for covariates (age and BMI). Linear regression analysis was performed to evaluate the FINDRISC as a predictor of changes in metabolic and clinical traits at the 4.6-year follow-up visit among those participants who had follow-up visit and who did not develop diabetes between the baseline and follow-up visits. Results are presented as unstandardized coefficients (B ± SE) in original units (one FINDRISC point), and standardized beta coefficients. Linear regression models were adjusted for age, corresponding trait at baseline, and follow-up time (in months). In a model including systolic and diastolic blood pressure (BP) the adjustment was additionally done for the use of antihypertensive medication at baseline. Cox regression was used to investigate the association of FINDRISC with incident type 2 diabetes (mean follow-up 8.2 years), drug-treated hypertension (mean follow-up time 6.0 years, participants with drug treatment for hypertension at baseline were excluded), CVD events and total mortality (mean follow-up 7.2 years) during the follow-up (a longer follow-up than for participants having follow-up re-examination is explained by the fact that for all participants the data are available from the National Drug Reimbursement registry and Mortality registry until December 31, 2013, and Hospital Discharge Registry). Hazard ratios (HRs) with their 95% confidence intervals (CI) are presented. The Cox regression models were adjusted for age, BMI, smoking and physical activity. After Bonferroni correction for multiple testing *P*<0.00125 was considered as statistically significant in linear regression models given 40 models tested, and *P*<0.0063 in Cox regression models (8 models tested). *P*<0.05 was considered as nominally significant.

## Results

### Baseline clinical and laboratory characteristics of the participants in the categories of the FINDRISC

All clinical and laboratory characteristics significantly differed across the four FINDRISC categories among 8,745 (FINRISC was missing for four participants) participants of the METSIM Study (Table 1). Category < 7 indicates low risk, category 7–11 slightly elevated risk, 12–14 category moderate risk, 15–20 high risk, and > 20 very high risk of diabetes. Clinical characteristics (age, BMI, waist, fat mass, systolic and diastolic BP), and laboratory measurements (FPG, 2hPG, Glucose AUC, TGs and ALT) increased across the FINDRISC categories, whereas HDL cholesterol, ApoA1, and adiponectin decreased. Insulin sensitivity (Matsuda ISI) and insulin secretion (Disposition index) significantly decreased with increasing points of the FINDRISC. The differences remained statistically significant after the adjustment for age and BMI.

**Table 1:** Clinical and metabolic characteristics of participants at the METSIM baseline study in the categories of Finnish Diabetes Risk Score (FINDRISC).

	FINDRISC categories (N = 8,745)									
	Category 1		Category 2		Category 3		Category 4			
	FINDRISC<7points (N = 2,011)		FINDRISC 7–11 points (N = 3,302)		FINDRISC 12–14 points (N = 1,615)		FINDRISC >15 points (N = 1,817)		*P* values	
Variables at baseline	N	Mean ± SD	N	Mean ± SD	N	Mean ± SD	N	Mean ± SD	*P*_overall_	P*_overall_
Age [years]	2,011	55.4±6.8	3,302	56.8±7.1	1,615	57.6±6.9	1,817	59.4±6.9	**<0.0001**	-
BMI [kg/m2]	2,011	24.0 ± 2.1	3,302	26.1±2.9	1,615	28.3±3.7	1,817	30.1±4.0	**<0.0001**	-
Waist circumference [cm]	2,011	88.5 ± 5.5	3,302	95.7±8.3	1,615	101.8±9.6	1,817	106.7±10.3	**<0.0001**	**<0.0001**
Fat mass [%]	2,008	19.2 ± 4.8	3,295	22.7±5.8	1,612	25.0±5.5	1,812	28.0±6.0	**<0.0001**	**<0.0001**
Systolic BP [mmHg]	2,011	132.7 ± 15.3	3,302	136.4±15.9	1,615	139.5±16.6	1,817	141.4±16.2	**<0.0001**	**<0.0001**
Diastolic BP [mmHg]	2,011	84.6 ± 8.7	3,302	86.8±9.0	1,615	88.9±9.6	1,817	89.3±9.2	**<0.0001**	**<0.0001**
LDL cholesterol [mmol/l]	2,011	3.39 ± 0.84	3,301	3.43±0.88	1,613	3.40±0.88	1,817	3.21±0.88	**<0.0001**	**<0.0001**
HDL cholesterol [mmol/l]	2,011	1.6 ± 0.4	3,302	1.5±0.4	1,613	1.4±0.4	1,817	1.3±0.4	**<0.0001**	**<0.0001**
Triglycerides [mmol/l]	2,011	1.13 ± 0.59	3,302	1.36±0.76	1,615	1.59±1.48	1,817	1.61±0.94	**<0.0001**	**<0.0001**
Apo A1[g/l]	2,011	1.46 ± 0.25	3,302	1.42±0.24	1,613	1.39±0.23	1,817	1.36±0.24	**<0.0001**	**0.0003**
Apo B[g/l]	2,011	0.99 ± 0.25	3,302	1.04±0.27	1,613	1.07±0.28	1,817	1.04±0.28	**<0.0001**	**<0.0001**
Adiponectin [μg/ml]	2,01	8.6 ± 4.8	3,301	7.9±4.1	1,615	7.7±.4.0	1,817	7.5±4.6	**<0.0001**	**0.0004**
ALT [U/l]	2,011	27.2 ± 21.0	3,302	30.1±17.3	1,615	34.3±22.2	1,817	35.5±21.0	**<0.0001**	**<0.0001**
FPG [mmol/l]	2,011	5.6 ± 0.4	3,302	5.7±0.5	1,615	5.8±0.5	1,817	5.9±0.5	**<0.0001**	**<0.0001**
2hPG [mmol/l]	2,011	5.4 ± 1.4	3,302	5.9±1.6	1,615	6.3±1.7	1,817	6.8±1.8	**<0.0001**	**<0.0001**
Glucose AUC	2,005	826.7 ± 122.7	3,289	872.4±130.0	1,611	913.4±129.6	1,81	956.2±133.2	**<0.0001**	**<0.0001**
Matsuda ISI [mg/dl, mU/l]	1,997	9.6 ± 4.4	3,283	7.3±4.1	1,608	5.6±3.2	1,805	4.5±2.7	**<0.0001**	**<0.0001**
Disposition index†	1,997	189.9 ± 81.0	3,283	170.0±69.6	1,608	151.3±63.0	1,805	133.6±58.6	<0.0001	**<0.0001**

Abbreviations: ALT, alanine aminotransferase; ApoA1, apolipoprotein A1; ApoB, apolipoprotein B; AUC, area under the curve; BMI, body mass index; BP, blood pressure FPG, fasting plasma glucose; FINDRISC, the Finnish Diabetes Risk Score; HDL, high-density lipoprotein; 2hPG, 2-hour plasma glucose; ISI, insulin sensitivity index; LDL, low-density lipoprotein; SD, standard deviation. *P* values were calculated with log-transformed variables (except for age), and were obtained from one-way ANOVA. ANCOVA was used to adjust for age and BMI. Significant P values (P<0.00125) are marked by bold font. *P*, unadjusted; P*, adjusted for age and BMI.

^†^Disposition index was calculated as Matsuda ISI x InsulinAUC_0-30_/GlucoseAUC_0-30_.

### Insulin sensitivity and secretion in the categories of the FINDRISC at baseline and follow-up studies

**Fig 1:**
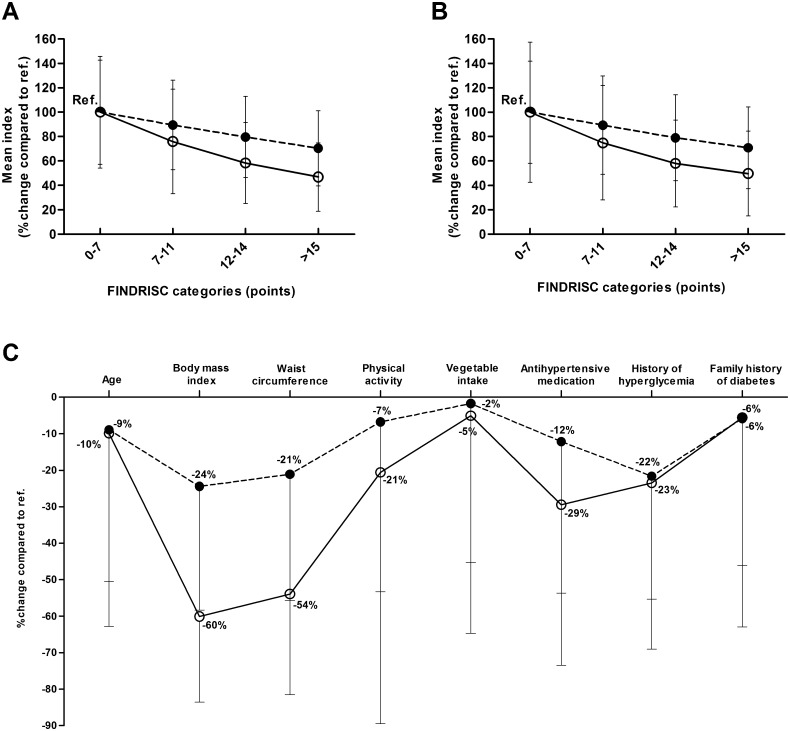
Percentage decreases in insulin sensitivity (Matsuda ISI, open circles) and insulin secretion (Disposition index, filled circles, dashed lines) across the Finnish Diabetes Risk Score (FINDRISC) categories compared to the reference category (< 7 points). **(A)** in the non-diabetic participants of the METSIM baseline study, **(B)** in the participants of the METSIM 4.6-year follow-up study (participants without diabetes or with newly-diagnosed diabetes at the follow-up examination) **(C)** in the subcategories of the eight components of the FINDRISC at baseline (reference categories in C for each component respectively are as follows: age<55 years, BMI<25 kg/m^2^, waist<94 cm, physical activity at least 30 min ≥2 times per week, vegetable intake every day, no antihypertensive medication, no history of hyperglycemia, no family history of diabetes). Data are means ± SD.

The markers of insulin sensitivity (Matsuda ISI) and insulin secretion (Disposition index) across the four FINDRISC categories at the METSIM baseline and follow-up studies are shown in Fig 1. Category 1 (FINDRISC <7 points) was set as the reference category. The Matsuda ISI and Disposition index significantly decreased across the FINDRISC categories at both baseline (Fig 1A) and follow-up (Fig 1B). The Matsuda ISI decreased by 53% and 50% in the baseline and follow-up studies (> 15 points), correspondingly, compared to the reference category. Similarly, the Disposition index significantly decreased by 30% and 29% in the baseline and follow-up studies, correspondingly, compared to the reference category. Fig 1C shows the percentage decreases in insulin secretion and insulin sensitivity between the extreme categories of each component of the FINDRISC in the cross-sectional study. Overall, decreases in the Matsuda ISI were larger than those for the Disposition index. The largest decreases in insulin sensitivity and insulin secretion were observed for the BMI component (Matsuda ISI was 60% lower and the Disposition index was 24% lower in the BMI>30 kg/m^2^ category than in the BMI<25 kg/m^2^ category), and the waist circumference component (Matsuda ISI was 53% lower and the Disposition index was 21% lower in the waist>102 cm category than in the waist<94 cm category), and the smallest decreases for vegetable intake, family history of diabetes and age (≤ 10%).

### Associations of the FINDRISC with clinical and metabolic traits at the follow-up visit

The FINDRISC was significantly associated with increases in BMI, waist circumference, fat mass, systolic and diastolic BP, glycemia (FPG, 2hPG, Glucose AUC), TGs, and ALT, and with decreases in LDL cholesterol, HDL cholesterol, ApoA, and eGFR during the follow-up (Table 2). Particularly strong associations were observed between the FINDRISC and increases in waist circumference, BMI, fat mass and glycemia (beta≥0.26), and decreases in insulin sensitivity (Matsuda ISI) and insulin secretion (Disposition index) (beta≤-0.27). After the adjustment for age, follow-up time and for corresponding trait at baseline most of the associations remained statistically significant except for BMI, systolic and diastolic BP.

**Table 2:** Association of the Finnish Diabetes Risk Score (FINDRISC) with metabolic and clinical traits in 5,401 participants without diabetes or with newly diagnosed type 2 diabetes at the METSIM follow-up visit (linear regression analysis).

	Effect size per FINDRISC point					
Variables at the 4.6-year follow-up visit	N	B	SE	beta	*P*	P*
Age [years]	5,401	0.307	0.019	0.214	-	-
BMI [kg/m2]	5,401	0.417	0.009	0.539	**<0.0001**	0.414
Waist circumference [cm]	5,401	1.200	0.024	0.556	**<0.0001**	**0.0001**
Fat mass [%]	5,356	0.636	0.017	0.448	**<0.0001**	**<0.0001**
Systolic BP [mmHg]	5,399	0.560	0.044	0.170	**<0.0001**	0.084
Diastolic BP [mmHg]	5,399	0.191	0.025	0.103	**<0.0001**	0.286
LDL cholesterol [mmol/l]	5,401	-0.025	0.002	-0.139	**<0.0001**	<0.0001
HDL cholesterol [mmol/l]	5,401	-0.017	0.001	-0.214	**<0.0001**	**0.0006**
Triglycerides [mmol/l]	5,401	0.023	0.002	0.155	**<0.0001**	**<0.0001**
Apo A1[g/l]	5,107	-0.006	0.001	-0.133	**<0.0001**	**<0.0001**
Apo B[g/l]	5,107	-0.001	0.001	-0.016	0.215	0.063
Adiponectin [μg/ml]	1,303	-0.072	0.029	-0.068	0.002	0.035
ALT [U/l]	5,401	0.405	0.047	0.116	**<0.0001**	**0.0001**
FPG [mmol/l]	5,401	0.030	0.002	0.257	**<0.0001**	**<0.0001**
2hPG [mmol/l]	5,401	0.109	0.006	0.259	**<0.0001**	**<0.0001**
Glucose AUC [mmol/l * min]	5,37	9.195	0.417	0.288	**<0.0001**	**<0.0001**
Matsuda ISI [mg/dl, mU/l]	5,345	-0.340	0.011	-0.378	**<0.0001**	**<0.0001**
Disposition index†	5,345	-4.189	0.206	-0.268	**<0.0001**	**<0.0001**
eGFR	5,401	1.092	0.007	0.221	**<0.0001**	**<0.0001**

Abbreviations: AUC, area under the curve; B (SE), unstandardized regression coefficient; beta, standardized regression coefficient; eGFR, estimated glomerular filtration rate, FPG, fasting plasma glucose; ISI; insulin sensitivity index; 2hPG, 2-hour plasma glucose. Effect sizes (B, SE) are given in the original units of the dependent variable per 1 point of the FINDRISC score, beta coefficient is standardized. *P* values were calculated using logarithmically transformed depended variables (except for age). *P*<0.00125 was considered as statistically significant (bold font), and *P*<0.05 as nominally significant (underlined).

^†^Disposition index was calculated as: Matsuda ISI x InsulinAUC_0-30_/GlucoseAUC_0-30_.

*P*, unadjusted. P*, adjusted for age, follow-up time (in months), and corresponding trait at baseline. Systolic and diastolic blood pressure was additionally adjusted for antihypertensive medication at baseline. The mean length of the follow-up was 4.6 years.

### Associations of the FINDRISC with overweight and obesity at METSIM baseline and follow-up studies

We additionally evaluated the associations of the FINDRISC with overweight and obesity at both baseline and follow-up studies S1 Table). The FINDRISC was significantly (*P*<0001) associated with overweight (BMI≥25 kg/m^2^), and obesity (BMI≥30 kg/m^2^) in both baseline and follow-up studies. The associations of the FINDRISC with obesity were stronger than the associations with overweight.

### The FINDRISC as a predictor for incident type 2 diabetes, drug-treated hypertension, cardiovascular (CVD) events, and total mortality in the follow-up study

The FINDRISC as a continuous variable significantly associated with an increased risk of incident type 2 diabetes (HR = 1.18, 95% CI 1.17–1.20), drug-treated hypertension (HR = 1.10, 95% CI 1.07–1.13), CVD events (HR = 1.05, 95% 1.03–1.07), and total mortality (HR = 1.05, 95% CI 1.03–1.08) (Table 3). After the adjustment for age, BMI, smoking and physical activity, the FINDRISC remained significantly associated with incident type 2 diabetes and drug-treated hypertension, but not with CVD events and total mortality. The FINDRISC as a categorical variable (≥12 vs. < 12 points) significantly associated with the risk of type 2 diabetes (HR = 4.14, 95% CI 3.51–4.89), drug treated hypertension (HR = 2.43, 95% CI, 1.87–3.15), CVD events (HR = 1.61, 95% CI 1.30–1.98), and total mortality (HR = 1.55, CI 1.27–1.89) when compared to the reference category (<12 points). The FINDRISC remained significantly associated with incident type 2 diabetes, drug-treated hypertension, and nominally with CVD events but not with total mortality after the adjustment for confounding factors (age, BMI, smoking and physical activity).

**Table 3:** The association of the Finnish Diabetes Risk Score (FINDRISC) with incident type 2 diabetes, new drug-treated hypertension, cardiovascular (CVD) events and total mortality in the METSIM follow-up study.

	FINDRISC as a continuous variable						FINDRISC as a categorical variable (≥12 points vs. <12 points)			
	N									
Outcome	Event	Total	HR	95% CI	*P*	P*	HR	95% CI	*P*	P*
Type 2 diabetes	693	8,745	1.18	1.17–1.20	**<0.0001**	**<0.0001**	4.14	3.51–4.89	**<0.0001**	**<0.0001**
Drug treatment for hypertension	225	7,169	1.10	1.07–1.13	**<0.0001**	**<0.0001**	2.43	1.87–3.15	**<0.0001**	**0.0001**
CVD events	351	8,196	1.05	1.03–1.07	**<0.0001**	0.063	1.61	1.30–1.98	**<0.0001**	0.015
Total mortality	392	8,745	1.05	1.03–1.08	**<0.0001**	0.532	1.55	1.27–1.89	**<0.0001**	0.516

Abbreviations: CI, confidence interval; HR, hazard ratio; N, number of events and participants. *P*, unadjusted, P*adjusted for age, BMI, smoking and physical activity. HRs and their 95% confidence intervals were obtained from Cox regression analyses. Participants with type 1 diabetes (N = 25), type 2 diabetes (N = 763), newly diagnosed type 2 diabetes at baseline (N = 649), and participants with myocardial infarction, stroke or receiving antihypertensive prior the baseline study were excluded from the analyses. *P*<0.0063 was considered as statistically significant (bold font). The mean length of follow-up was as follows: 8.2 years for incident type 2 diabetes, 6.0 years for new drug-treated hypertension, and 7.2 years for CVD events and total mortality.

## Discussion

In our follow-up study of the large population-based METSIM cohort we evaluated the associations of the FINDRISC with changes in insulin secretion, insulin sensitivity, insulin resistance related traits and the risk of type 2 diabetes, drug-treated hypertension, CVD events, and total mortality. We hypothesized that the FINDRISC is likely to be a marker of impaired insulin secretion since insulin secretion defect is needed for the conversion to diabetes. Indeed, our results demonstrated for the first time that the FINDRISC was significantly associated with impairment in insulin secretion and insulin sensitivity, and adverse changes in insulin resistance related traits (increases in BMI, waist, fat percentage, obesity, overweight, TGs, blood pressure, and incident drug-treated hypertension). We also confirmed that the FINDRISC was significantly associated with the risk of incident type 2 diabetes, CVD events, and total mortality [15,26,27].

Impaired insulin secretion and insulin resistance are the two major pathophysiological defects in type 2 diabetes. Our findings that the FINDRISC predicts changes in insulin secretion and insulin sensitivity gives evidence that this risk score reflects both major pathophysiological defects needed for the conversion to type 2 diabetes. All eight components included in the calculation of the FINDRISC were associated more strongly with insulin resistance than with impaired insulin secretion. In agreement with this finding are our results showing that the FINDRISC was also associated with adverse changes in insulin resistance related traits (increases in BMI, waist, fat percentage) in our follow-up study of the METSIM cohort. Decreases is insulin sensitivity and insulin secretion were largest for BMI and waist circumference suggesting that these two obesity markers in the FINRISC are likely to be the most important drivers for the conversion to diabetes.

The FINDRISC or its modifications have been applied in cross-sectional and prospective studies as a tool to evaluate the risk of diabetes or hyperglycemia [28–30]. In previous studies the FINDRISC has also predicted coronary heart disease, stroke and total mortality [31,32]. However, the predictive value of the FINDRISC for future drug-treated hypertension has not been previously investigated. We demonstrated that the FINDRISC as a continuous and categorical variable is a significant predictor for future drug-treated hypertension, in addition to incident type 2 diabetes and CVD events. The ability of the FINDRISC to predict drug-treated hypertension was stronger than that of CVD events. This is likely explained by a strong association of elevated blood pressure with insulin resistance [33,34].

The strength of our study is a large sample size and a long follow-up period. The limitation of our study is that it included only middle-aged and elderly Finnish men, and therefore studies in woman, other populations and ethnic groups are needed to confirm our findings. Furthermore, we applied validated surrogate markers of insulin sensitivity and insulin secretion. Although direct measurements of insulin sensitivity and insulin secretion are more accurate, the large sample size of our study did not allow us to perform these measurements.

In conclusion, we demonstrated that the FINDRISC is a simple tool for the detection of early adverse changes in insulin secretion and insulin sensitivity which likely explain the conversion to type 2 diabetes. We also showed that the FINDRISC predicts adverse changes in insulin resistance related traits and the risk of drug-treated hypertension which may contribute to elevated risk of CVD events.

## Supporting Information

S1 Table Associations of the Finnish Diabetes Risk Score (FINDRISC) with overweight and obesity in the METSIM baseline and follow-up studies (linear regression analysis). Abbreviations: B (SE), unstandardized regression coefficient; beta, standardized regression coefficient; FINDRISC, the Finnish Diabetes Risk Score. *P*, unadjusted; P*, adjusted for age, and follow-up analysis also for follow-up time.
(DOCX)
