# Supplementary material for: Finnish Diabetes Risk Score Is Associated with Impaired Insulin Secretion and Insulin Sensitivity, Drug-Treated Hypertension and Cardiovascular Disease: A Follow-Up Study of the METSIM Cohort
Source: PLoS One. 2016 Nov 16;11(11):e0166584. doi: 10.1371/journal.pone.0166584 (PMC5112858; doi:10.1371/journal.pone.0166584)
Supplement: S1 Table — Abbreviations: B (SE), unstandardized regression coefficient; beta, standardized regression coefficient; FINDRISC, the Finnish Diabetes Risk Score. P, unadjusted; P*, adjusted for age, and follow-up analysis also for follow-up time. (DOCX) [file pone.0166584.s001.docx]

|  | **FINDRISC** | | | | | | | |
| --- | --- | --- | --- | --- | --- | --- | --- | --- |
|  | **Effect size per one FINDRISC point** | | | | | | | |
|  | **N** | **B** | **SE** | **beta** | ***P*** | | ***P**** | |
|  | **Baseline study** | | | | | | | |
| Overweight (BMI≥25 kg/m^2^) | 4,304 | 0.082 | 0.005 | 0.242 | **<0.0001** | | **<.0.001** | |
| Obese (BMI≥30 kg/m^2^) | 1,499 | 0.106 | 0.021 | 0.127 | **<0.0001** | | **0.001** | |
|  | **Follow-up study** | | | | | | | |
| Overweight (BMI≥25 kg/m^2^) | 2,680 | 0.085 | 0.006 | 0.269 | **<0.0001** | **<0.0001** | |  |
| Obese  (BMI≥30 kg/m^2^) | 872 | 0.160 | 0.024 | 0.224 | **<0.0001** | | **<0.0001** | |
